# Rathke's cleft cyst apoplexy presented as pituitary apoplexy: A case report

**DOI:** 10.1016/j.ijscr.2024.110571

**Published:** 2024-11-09

**Authors:** Soumya Pahari, Yugant Khand, Stuti Yadav, Paawan Bahadur Bhandari, Purushottam Baniya, Nishan Bhandari

**Affiliations:** a Nepalese Army Institute of Health Sciences, College of Medicine, Sanobharyang, 44600 Kathmandu, Nepal; b Shree Birendra Hospital, Chhauni, Kathmandu 44600, Nepal

**Keywords:** Rathke cleft cyst, Pituitary apoplexy, Transsphenoidal surgery, Case report

## Abstract

**Introduction and importance:**

Rathke's cleft cyst (RCC) are non-malignant lesions arising from remnants of Rathke's pouch in the pituitary gland. Apoplexy is seen commonly in pituitary macroadenomas but very rarely in RCCs.

**Case presentation:**

A 30-year-old male presented with a severe headache and vomiting. Imaging revealed an enlarged pituitary gland with possible hemorrhage in a RCC. The cyst was evacuated with endonasal transsphenoidal surgery, and histology confirmed RCC. Follow-up imaging showed complete cyst resolution, although he required levothyroxine for hypothyroidism.

**Clinical discussion:**

The occurrence of hemorrhage within RCCs, although rare, mimics pituitary tumor apoplexy often complicating the diagnosis. The management is similar to pituitary tumor apoplexy, primarily involving surgical intervention. Postoperative care may require long-term hormonal replacement therapy in a significant number of patients.

**Conclusion:**

This report underscores the importance of considering RCC in the differential diagnoses of pituitary lesions with hemorrhage with promising surgical outcome.

## Highlights

• Rathke's cleft cyst are non-malignant lesions arising from remnants of Rathke's pouch in the pituitary gland.
• The rare occurrence of hemorrhage within RCCs mimics pituitary tumor apoplexy often complicating the diagnosis.
• The management involves surgical intervention with postoperative hormonal dysfunction occurring rarely.

## 1 Introduction

Rathke's cleft cyst (RCC) are benign cystic lesion within the sella turcica arising from epithelial remnants of Rathke's pouch [1,2]. RCC are mostly asymptomatic but sometimes they enlarge and can cause compressive effects on surrounding structures like pituitary gland and optic chiasma, making it symptomatic [1,3]. The common manifestations of symptomatic RCC are headaches, endocrinopathies, and visual impairment [1,3]. Very rarely, RCC can present with acute symptoms resembling pituitary apoplexy [1,[3], [4], [5]]. Apoplexy is seen in around 10.4 % of pituitary macroadenoma, 0.3 % in microadenoma, rarely in pineal tumors, and very rarely in RCC [6]. Here, we present a case of a 30-year-old male patient with RCC presenting as apoplexy.

This work has been reported in line with the SCARE 2023 guideline [12].

## 2 Case presentation

**Fig. 1:**
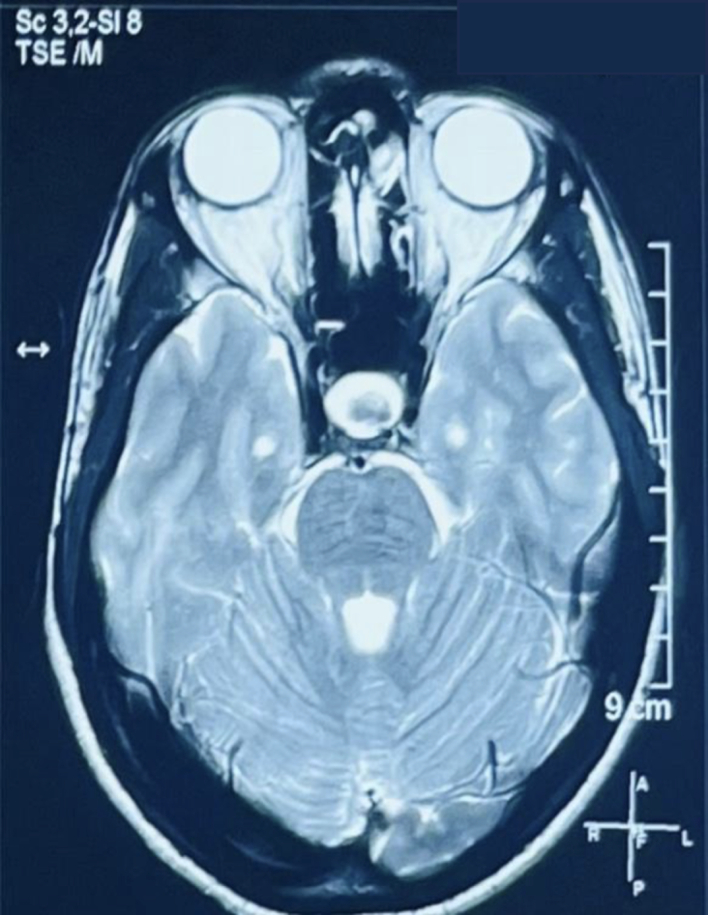
MR imaging of the brain showing that the pituitary gland is enlarged measuring about 13 mm (CC) × 13.2 mm (AP) × 16 mm (transverse), with a low T2 signal nodule measuring 5.6 × 9.5 mm seen within.

**Fig. 2:**
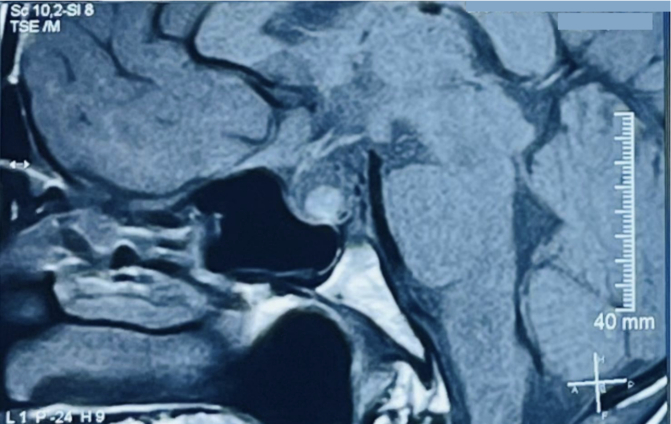
On the post-gadolinium images, there was peripheral rim enhancement of the gland with no enhancement in the majority of the central gland.

**Fig. 3:**
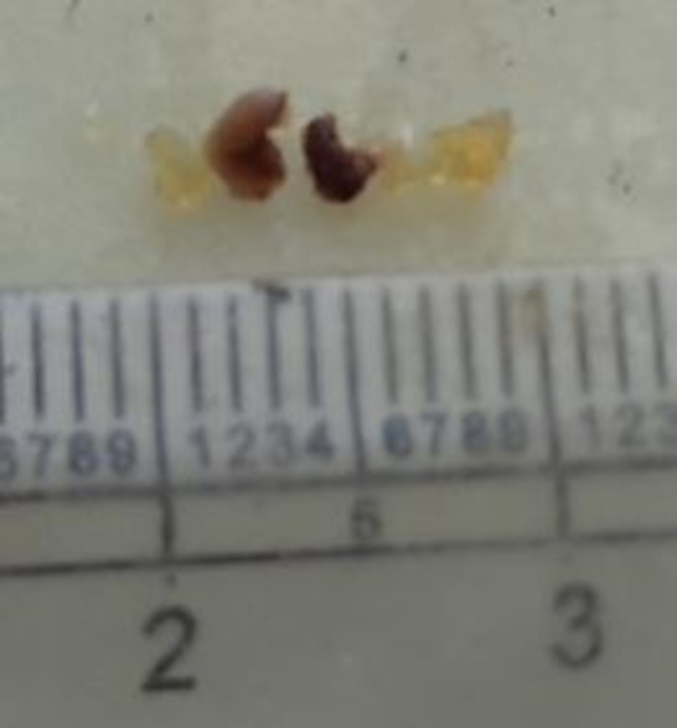
Multiple pieces of greyish yellow to dark brown tissue measuring 1.0 × 0.2 × 0.1 cm when aligned together. (For interpretation of the references to colour in this figure legend, the reader is referred to the web version of this article.)

**Fig. 4:**
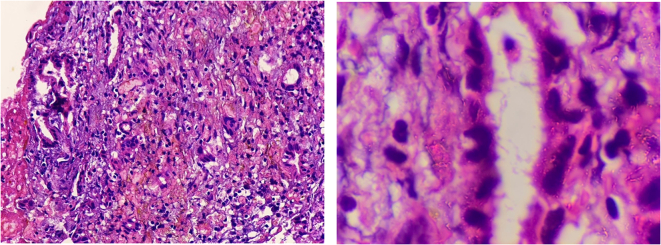
Histopathology examination shows few glands with occasional ciliated epithelium scattered in a hemorrhagic fibrous and fibrino-inflammatory background. Dense pink mucinous material is associated. (For interpretation of the references to colour in this figure legend, the reader is referred to the web version of this article.)

A 30-year-old male with no known comorbidities presented to the emergency department with a sudden onset of a headache that had progressively intensified over three days, accompanied by multiple episodes of non-projectile vomiting. There was no history of trauma, loss of consciousness, and abnormal body movements. He had no significant medical history, family history, or similar history in the past. On examination, the vital signs were within normal limits. A complete neurological examination including cranial nerves was unremarkable. The rest of the physical examination was unremarkable. Laboratory results revealed mild hyponatremia. A non-contrast computerized tomography (NCCT) of the head revealed an enlarged pituitary gland measuring about 13 mm (CC) × 13.2 mm (AP) × 16 mm (transverse). Magnetic resonance imaging (MRI) showed a low T2 signal nodule measuring 5.6 × 9.5 mm within the gland. Fig. 1 On the post‑gadolinium images, there was peripheral rim enhancement of the gland with no enhancement in the majority of the central gland with effacement of the pituitary stalk. Fig. 2 These findings imply the potential occurrence of hemorrhage in a pituitary adenoma, Rathke cleft cyst (RCC), or craniopharyngioma. A surgical exploration was planned with transsphenoidal microsurgery which revealed a bulging of the sella floor. Upon opening the dura mater, there was an immediate expression of a bloody serous, mucinous, and yellowish substance. Cyst contents were completely evacuated and the cyst wall was partially excised. Fig. 3 On histological examination, the lesion was a sellar cyst with epithelial linings which was suggestive of RCC, as well as evidence of acute hemorrhage mixed in with the contents of the cyst. Fig. 4 Postoperatively, the patient's headaches improved. The patient developed secondary adrenal deficiency post operatively which resolved 2 months after the operation.

At 3 months follow-up, the patient was asymptomatic and repeat imaging revealed complete resolution of the cyst. There were no changes in his hormonal assay except for an isolated increase in thyroid stimulating hormone (TSH value: 8 mU/L) and negative antithyroid peroxidase antibodies. His six weeks and 12 weeks follow-up showed no new changes.

## 3 Clinical discussion

Rathke's cleft cysts (RCCs) are non-malignant lesions that originate from epithelial remnants of Rathke's pouch, a developmental structure in the pituitary gland [1]. RCCs primarily occur in the sellar and suprasellar regions of the brain and can range from a few millimeters to 1–2 cm [2,3]. These cysts demonstrate a higher prevalence within the age range of 30 to 50 years, with the mean age of presentation being 36.87 years, indicating a young mean age of presentation [2,7].

RCC is a rare occurrence and has been sparsely documented in the scientific literature with a limited number of case reports [7]. RCC can rarely present with hemorrhage within the cyst, a distinct clinical manifestation that resembles the clinical syndrome of pituitary tumor apoplexy [8]. This unique presentation adds complexity to the differential diagnosis and requires careful evaluation to differentiate between the two conditions.

Headache, specifically paroxysmal headache localized in the forehead, is the most frequent clinical manifestation observed in patients with symptomatic RCC [9] The other common clinical presentation of RCC apoplexy includes visual disturbances, hormonal dysfunction, and neurological deficits [1,3]. These symptoms arise due to the compression and impairment of the pituitary gland, optic chiasm, or adjacent brain structures by enlarged RCC [9,10]. Hyperprolactinemia is the most frequently observed disorder of the hypothalamic-pituitary axis, with hypogonadism, hypocortisolism, hypothyroidism, and diabetes insipidus following as other commonly affected conditions [7].

The use of non-contrast computed tomography (NCCT) can be inconclusive to differentiate pituitary mass from RCC like in our case. The diagnosis of RCC apoplexy is typically made through neuroimaging techniques, such as magnetic resonance imaging (MRI), but according to a previous study, approximately 50 % of surgically confirmed RCCs were misdiagnosed initially as pituitary adenomas [11]. The clinical, radiographic, and surgical characteristics may contribute to the differential diagnosis of RCC apoplexy, but the confirmatory diagnosis of RCC apoplexy can be made only on histopathological confirmation [1,7]. RCC is histologically characterized by the presence of cuboidal or columnar epithelium containing goblet cells and cilia [1,2]. RCC with hemorrhage typically presents with distinct histological features, including the presence of hemosiderin pigment and infiltration of lymphocytes and plasma cells [7].

The underlying cause of bleeding into an RCC remains unclear. Recent reports suggest that RCC apoplexy may be attributed to hemorrhage originating from compressed portal veins or newly formed delicate vessels within the granulation tissue of the cyst wall, particularly in larger cysts [1,7]. Since RCCs are lined by a fragile single layer of cuboidal or columnar epithelium including cilia and goblet cells, they are highly susceptible to rupture following minimal fluctuation in arterial pressure [1,2].

Distinguishing RCCs from other sellar lesions can pose challenges in MRI imaging due to their similar appearances [7]. The signal intensities exhibited by an RCC on T1 and T2-weighted images can vary depending on its cystic content and the presence of an associated intracystic nodule [2,11]. Typically, this nodule presents as a region within the cyst that demonstrates T1-weighted hyperintensity, T2-weighted hypointensity and lacks gadolinium enhancement [1]. Furthermore, RCC and cystic pituitary adenoma can be differentiated by examining the presence of a fluid-fluid level, septation, off-midline location, and the existence of an intracystic nodule [11].

The management approach for patients presenting with sudden bleeding into an RCC closely resembles that of individuals experiencing pituitary tumor apoplexy [1,7]. Surgical intervention is the established therapeutic approach for the treatment of RCC apoplexy [9]. Endoscopic endonasal transsphenoidal approach (EETA) has emerged as a standard treatment modality for symptomatic RCCs, offering potential benefits such as reduced surgical trauma and improved postoperative recovery rates [9]. The presence of epithelial squamous metaplasia in the histopathological examination can serve as a predictive factor for recurrence following surgical resection [7]. Postoperatively, a substantial proportion of patients, up to 24 %, necessitate long-term hormonal replacement therapy [7].

## 4 Conclusion

This report underscores the importance of considering RCC in the differential diagnoses of pituitary lesions with hemorrhage with promising surgical outcome. Although rare, hemorrhage in RCC adds complexity to the diagnosis due to its resemblance to other pituitary lesions on MRI. Effective management relies on precise diagnosis with imaging, histopathology, and timely surgical intervention.

## Consent

Written informed consent was obtained from the patient for publication of this case report and accompanying images. A copy of the written consent is available for review by the Editor-in-Chief of this journal on request.

## Ethical approval

The case report is exempt from ethical approval in our institution.

## Guarantor

Yugant Khand.

## Research registration number

Not applicable.

## Funding

This research did not receive any specific grant from funding agencies in the public, commercial, or not-for-profit sectors.

## Provenance and peer review

Not commissioned, externally peer-reviewed.

## Author contribution

All the authors contributed in conceptualizing, writing and editing the manuscript.

## Conflict of interest statement

All authors declare that they have no conflict of interest.

## References

[1] Kim E. (2012). A Rathke’s cleft cyst presenting with apoplexy. J. Korean Neurosurg. Soc..

[2] Larkin S., Karavitaki N., Ansorge O. (2014). Rathke’s cleft cyst. Handb. Clin. Neurol..

[3] Shatri J., Ahmetgjekaj I. (2018). Rathke’s cleft cyst or pituitary apoplexy: a case report and literature review. Open Access Maced. J. Med. Sci..

[4] Jung H.N., Kim S.T., Kong D.S., Suh S.I., Ryoo I. (2020). Rathke cleft cysts with apoplexy-like symptoms: clinicoradiologic comparisons with pituitary adenomas with apoplexy. World Neurosurg..

[5] Constantinescu S.M., Wilms G., Furnica R.M., Duprez T., Maiter D. (2022). Conservative management of complicated Rathke’s cleft cyst mimicking pituitary apoplexy. Endocrinol. Diabetes Metab. Case Rep..

[6] Shah R., Waghmare P.Y., Babhulkar S., Sonkusare S. (2022). Rathke’s cleft cyst apoplexy-a newly introduced terminology with presentation of seizure. Neurol. India.

[7] Elarjani T., Alhuthayl M.R., Dababo M., Kanaan I.N. (2021). Rathke cleft cyst apoplexy: hormonal and clinical presentation. Surg. Neurol. Int..

[8] Chaiban J.T., Abdelmannan D., Cohen M., Selman W.R., Arafah B.M. (2011). Rathke cleft cyst apoplexy: a newly characterized distinct clinical entity. J. Neurosurg..

[9] Tang C., Wang P., Liu J., Jiang H., Zhang G., Wu N. (2022). Endoscopic endonasal transsphenoidal approach for symptomatic Rathke cleft cyst: a case series. Exp. Ther. Med..

[10] Brou C., Tatar I.G. (2023). Different faces of Rathke’s cleft cyst. J. Belg. Soc. Radiol..

[11] Park M., Lee S.K., Choi J. (2015). Differentiation between cystic pituitary adenomas and Rathke cleft cysts: a diagnostic model using MRI. AJNR Am. J. Neuroradiol..

[12] Sohrabi C., Mathew G., Maria N., Kerwan A., Franchi T., Agha R.A., Collaborators (May 1 2023). The SCARE 2023 guideline: updating consensus surgical CAse REport (SCARE) guidelines. Int. J. Surg..

